# Assessing the Implementation and Effectiveness of the Electronic Patient-Reported Outcome Tool for Older Adults With Complex Care Needs: Mixed Methods Study

**DOI:** 10.2196/29071

**Published:** 2021-12-02

**Authors:** Carolyn Steele Gray, Edward Chau, Farah Tahsin, Sarah Harvey, Mayura Loganathan, Brian McKinstry, Stewart W Mercer, Jason Xin Nie, Ted E Palen, Tim Ramsay, Kednapa Thavorn, Ross Upshur, Walter P Wodchis

**Affiliations:** 1 Bridgepoint Collaboratory for Research and Innovation Lunenfeld-Tanenebaum Research Institute Sinai Health Toronto, ON Canada; 2 Institute of Health Policy, Management and Evaluation Dalla Lana School of Public Health University of Toronto Toronto, ON Canada; 3 Logibec Inc (QoC Health Inc) Toronto, ON Canada; 4 Ray D Wolfe Department of Family Medicine Mount Sinai Hospital Toronto, ON Canada; 5 Department of Family and Community Medicine Faculty of Medicine University of Toronto Toronto, ON Canada; 6 Usher Institute College of Medicine and Veterinary Medicine University of Edinburgh Edinburgh United Kingdom; 7 Institute for Better Health Trillium Health Partners Mississauga, ON Canada; 8 Institute for Health Research Kaiser Permanente Colorado Denver, CO United States; 9 Clinical Epidemiology Program Ottawa Hospital Research Institute Ottawa, ON Canada; 10 School of Epidemiology and Public Health University of Ottawa Ottawa, ON Canada

**Keywords:** older adults, goal-oriented care, quality of life, self-management, primary care, eHealth, pragmatic trial, mobile phone

## Abstract

**Background:**

Goal-oriented care is being adopted to deliver person-centered primary care to older adults with multimorbidity and complex care needs. Although this model holds promise, its implementation remains a challenge. Digital health solutions may enable processes to improve adoption; however, they require evaluation to determine feasibility and impact.

**Objective:**

This study aims to evaluate the implementation and effectiveness of the electronic Patient-Reported Outcome (ePRO) mobile app and portal system, designed to enable goal-oriented care delivery in interprofessional primary care practices. The research questions driving this study are as follows: Does ePRO improve quality of life and self-management in older adults with complex needs? What mechanisms are likely driving observed outcomes?

**Methods:**

A multimethod, pragmatic randomized controlled trial using a stepped-wedge design and ethnographic case studies was conducted over a 15-month period in 6 comprehensive primary care practices across Ontario with a target enrollment of 176 patients. The 6 practices were randomized into either early (3-month control period; 12-month intervention) or late (6-month control period; 9-month intervention) groups. The primary outcome measure of interest was the Assessment of Quality of Life-4D (AQoL-4D). Data were collected at baseline and at 3 monthly intervals for the duration of the trial. Ethnographic data included observations and interviews with patients and providers at the midpoint and end of the intervention. Outcome data were analyzed using linear models conducted at the individual level, accounting for cluster effects at the practice level, and ethnographic data were analyzed using qualitative description and framework analysis methods.

**Results:**

Recruitment challenges resulted in fewer sites and participants than expected; of the 176 target, only 142 (80.6%) patients were identified as eligible to participate because of lower-than-expected provider participation and fewer-than-expected patients willing to participate or perceived as ready to engage in goal-setting. Of the 142 patients approached, 45 (32%) participated. Patients set a variety of goals related to self-management, mental health, social health, and overall well-being. Owing to underpowering, the impact of ePRO on quality of life could not be definitively assessed; however, the intervention group, ePRO plus usual care (mean 15.28, SD 18.60) demonstrated a nonsignificant decrease in quality of life (t_24_=−1.20; *P*=.24) when compared with usual care only (mean 21.76, SD 2.17)*.* The ethnographic data reveal a complex implementation process in which the meaningfulness (or coherence) of the technology to individuals’ lives and work acted as a key driver of adoption and tool appraisal.

**Conclusions:**

This trial experienced many unexpected and significant implementation challenges related to recruitment and engagement. Future studies could be improved through better alignment of the research methods and intervention to the complex and diverse clinical settings, dynamic goal-oriented care process, and readiness of provider and patient participants.

**Trial Registration:**

ClinicalTrials.gov NCT02917954; https://clinicaltrials.gov/ct2/show/NCT02917954

## Introduction

### Background

The rising population of older adults with multimorbidity and complex care needs requires that health systems adjust to meet this new demand [1,2]. Complex care needs of patients go beyond multimorbidity alone, as these individuals will experience biopsychosocial challenges and barriers that make it more difficult for them to manage their multiple chronic physical and mental illnesses [3,4]. Increasingly, digital health solutions are being adopted to support this patient population through tools that enable medication management [5], information sharing [6], care planning [7], chronic disease management and monitoring [8,9], and virtual care tools, particularly since the onset of the COVID-19 pandemic [10]. Of particular use to older adults with complex care needs are solutions that enable person-centered and holistic care delivery to better address their multiple health and social care needs [3,11-17]. Although a person-centered approach has been identified as a priority [16], organizations and providers continue to struggle with how to put the vision of person-centered care into practice [18].

Person-centered care may be operationalized by adopting a goal-oriented care (GOC) approach that involves eliciting patient-identified goals to drive care planning and decision-making [13,14,19,20]. Effectively, this model of care shifts from asking a patient “What is the matter with you?” to “What matters to you?” [21] From a patient perspective, GOC represents a more meaningful and holistic approach to care and decision-making [22]. Emerging studies of GOC report reduced treatment burden for patients with multiple chronic conditions [23] and reductions in acute inpatient days and mortality [24]. The pragmatic trial of the Health TAPESTRY program, a digitally enabled, community-based GOC program, evaluated the program’s impact on goal attainment, self-efficacy, quality of life, optimal aging, social support, empowerment, physical activity, falls, and access. The Health TAPESTRY trial demonstrated a shift from reactive to proactive care [25]; however, similar to many other studies of person-centered care [26,27], Health TAPESTRY did not demonstrate an impact on patient outcomes.

Among the challenges in evaluating an approach such as GOC, in particular a digitally enabled GOC model, is that it is a complex intervention that is delivered to a complex patient population within a complex system. Conventional methods, such as randomized controlled trials, that apply rigid methods and rely on assumptions of linear cause-effect perspectives [28] may result in *controlling* for the variables that we need to capture [29]. Greenhalgh and Papoutsi [28] instead suggest methods that adopt a systems mindset that allows for adaptability, iteration, and design-thinking better suited to capturing “changing interrelationships between parts of the system.” The evaluation presented in this paper adopts this systems mindset to evaluate the electronic Patient-Reported Outcome (ePRO) tool, a novel mobile device and a linked portal system that enables GOC delivery to older adults with complex care needs receiving care in interdisciplinary primary care practices. This evaluation is the latest iteration of a multiphase developmental evaluation of ePRO that took place in Ontario, Canada, from April 2018 to June 2019.

### Objective and Research Questions

**Figure 1:**
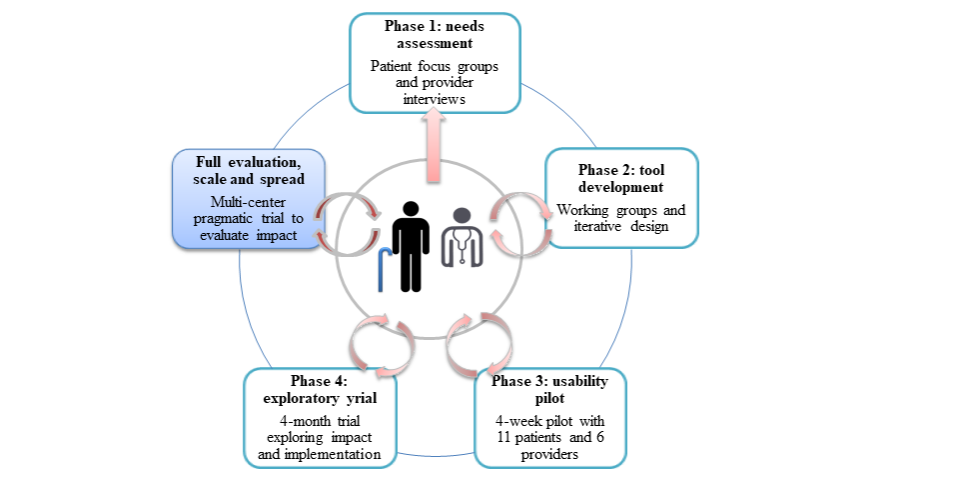
electronic Patient-Reported Outcome (ePRO) co-design steps.

This developmental evaluation incorporates a pragmatic, stepped-wedge, cluster trial with embedded ethnographic case studies, building on previous stages of design, development, and testing [30-33] (see Figure 1 for a visual representation of how this work builds on previous stages). This study expands the findings from our exploratory trial [33] as a means to engage in what Tsoukas terms “conjunctive theorizing to generate rich pictures of complex phenomena by drawing together different kinds of data from multiple sources” [34]. This work was guided by the following research questions:

1. Does ePRO improve quality of life and self-management in older adults with complex needs?
2. What mechanisms are likely driving observed outcomes?

Regarding the first research question, it is hypothesized that the ePRO tool will have a positive impact on quality of life and patients’ ability to self-manage.

## Methods

### Design

**Figure 2:**
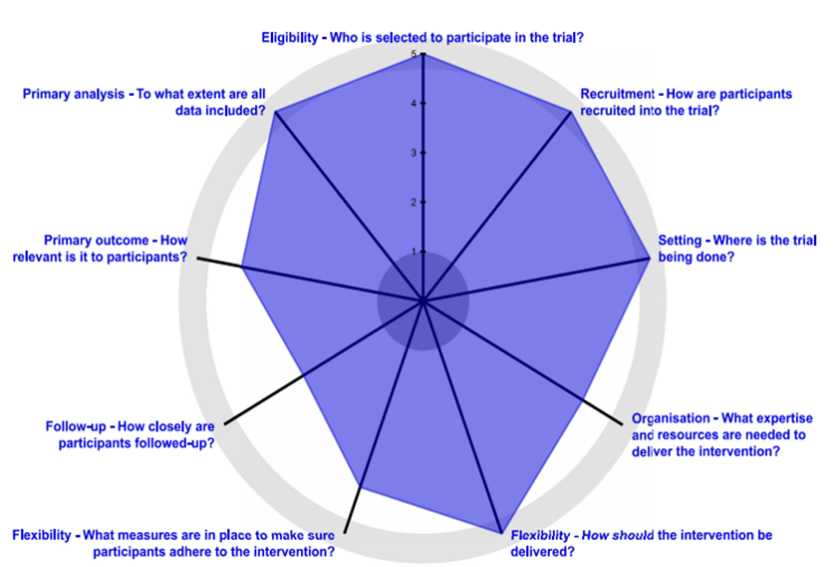
PRECIS-2 (Pragmatic Explanatory Continuum Indicator Summary) Wheel for electronic Patient-Reported Outcome (ePRO) trial.

Aligned with Medical Research Council guidelines for evaluating complex interventions [35], a developmental evaluation approach was applied to collect outcome, process, and context measures to support decision-making and technology modifications [36]. A pragmatic, stepped-wedge, cluster randomized trial design was used to assess the effectiveness of the ePRO tool [37]. The Pragmatic Explanatory Continuum Indicator Summary (PRECIS-2) wheel in Figure 2 (see Multimedia Appendix 1 for description of the wheel domains as related to this trial) describes the degree to which the trial represents a pragmatic design. This design was the most feasible and appropriate approach given the nature of the intervention, time, and resources available [38] and the desire to complete in a real-world setting [39]. An embedded ethnographic case study was included, aligned with the methods outlined by Greenhalgh and Swinglehurt [40] for evaluating complex technological innovations. The case studies offer rich contextual and process information that accounts for complex interrelationships between variables that are missed by looking at outcome data alone.

**Figure 3:**
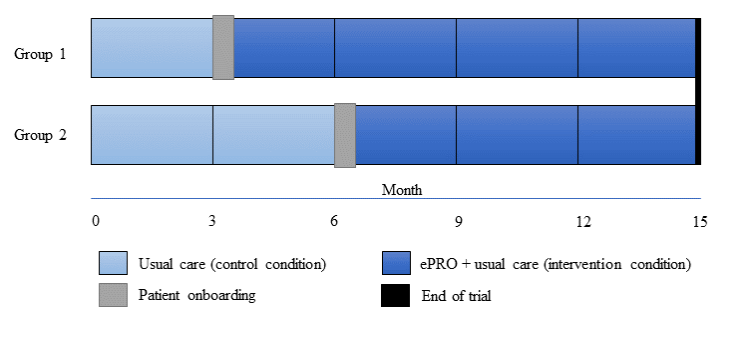
Stepped-wedge design for electronic Patient-Reported Outcome (ePRO) evaluation.

The trial was conducted in 6 comprehensive primary care practices called Family Health Teams (FHTs) across Ontario, Canada, over a 15-month period. Following the stepped-wedge design, all FHT sites started in the control period where all recruited patients received usual care (no change to their care delivery from the primary care team). A random number generator assigned sites to either the early intervention (n=3 sites) or late intervention (n=3 sites) groups. The early intervention group (group 1) was assigned to the intervention for 12 months after the initial 3-month control period. The FHTs in group 2 were switched to the intervention group for 9 months after a 6-month control period. Figure 3 shows a diagram of the stepped-wedge design.

### Intervention: The ePRO Tool

The ePRO tool was developed via multiphase user-centered co-design methods and represents an important divergence from many available systems that are focused on a single disease or are built to enhance existing provider-led models of care. The tool is designed to encourage a shift in the care process toward a person-centered model by enabling the full GOC process, including goal elicitation, ongoing monitoring, and goal modification [41]. Consistent with co-design methods, the tool was iteratively developed with input from patients with complex care needs, caregivers, and a multidisciplinary primary care team [30,31]. The tool has undergone usability testing [32] and an exploratory trial [33]. Findings from these studies were used to update and adapt the tool to user needs and different primary care settings. At the time of the trial, the ePRO tool did not connect to other existing technology systems, such as electronic medical records (EMRs) or other available platforms; however, the system was built so interoperability would be possible (see Multimedia Appendix 2 for wireframes).

### Population and Setting

**Figure 4:**
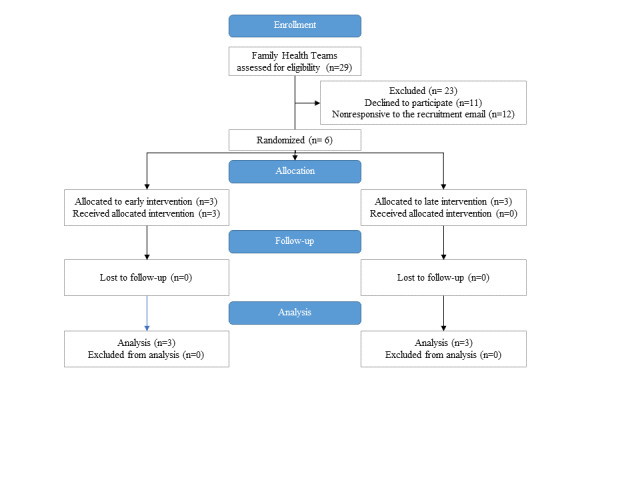
CONSORT (Consolidated Standard of Reporting Trials) flow diagram–Family Health Team (FHT) recruitment.

**Figure 5:**
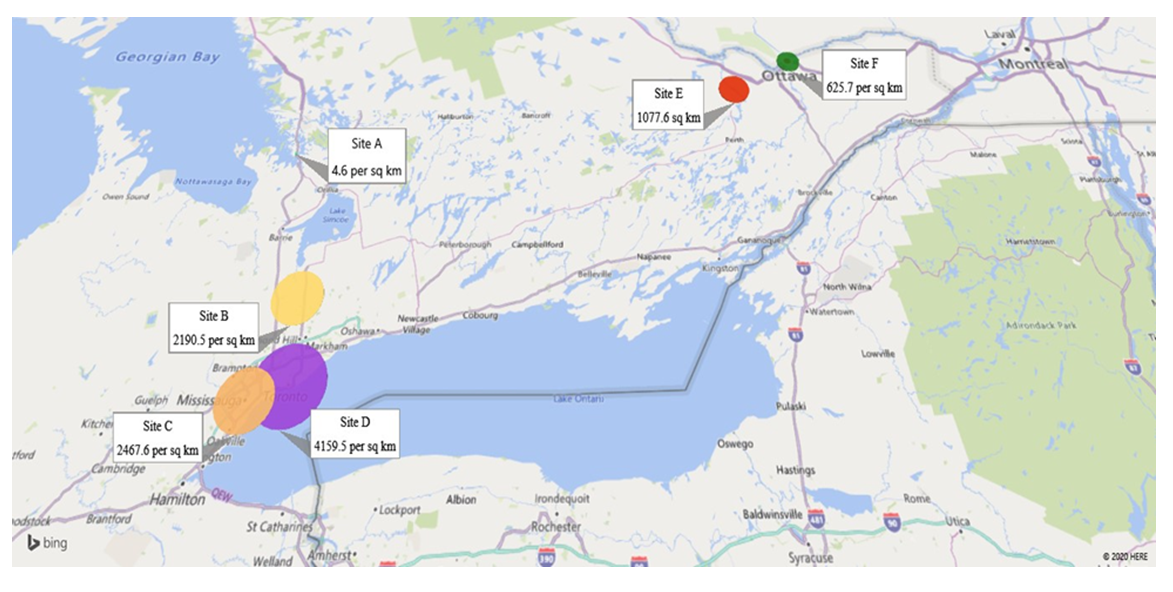
Population density across the regions (persons per square km). Source: Statistics Canada 2016 Census [43].

A 2-stage sampling strategy was implemented, first recruiting FHTs, followed by complex patients within each FHT. FHTs in Ontario are similar to Patient-Centered Medical Homes in the United States in that they both seek to provide comprehensive primary care services through a physician-led multidisciplinary team [42]. Working in collaboration with the project’s decision-making partner, the Association of Family Health Teams of Ontario (AFHTO; representing all 184 FHTs in Ontario), a multipronged FHT recruitment strategy was pursued, including (1) email invitations sent to AFHTO member sites, (2) a webinar session with AFHTO quality improvement specialists who could identify eligible sites, and (3) an information booth at the annual AFHTO conference (October 2016 in Toronto, Ontario) where study information was shared with delegates. From these avenues, 29 sites expressed interest to be assessed for eligibility, with 6 FHTs agreeing to participate (see the Figure 4 CONSORT flow diagram of site recruitment). As FHTs are geographically diverse, there is no chance of cross-contamination of providers across different sites. The characteristics of the participating sites, as compared with FHTs across Ontario, are summarized in Table 1, and the population densities of the regions are depicted in Figure 5. As can be seen in Figure 5, sites A and F were in rural settings, sites D and E were in urban settings, and sites B and C were medium urban as consistent with Statistics Canada definitions of rurality [43]. Approximately 36% (59/165) of FHTs are located in rural settings.

Providers eligible to participate in the study had to be provided care to patients rostered at the FHT. Providers can be employed full-time, part-time, or casual.

**Table 1:** Family Health Team characteristics^a^.

Characteristics		Group 1									Group 2										Ontario FHTs^b^, mean (SD)
		Site A			Site D			Site E			Site B			Site C			Site F				
		n (%)	N	n (%)		N	n (%)		N	n (%)		N	n (%)		N	n (%)		N			
**Number of providers enrolled in the ePRO^c^ study**																					
	Total number of providers	9 (69)	13	4 (22)		18	6 (27)		22	1 (6)		17	2 (17)		12	7 (54)		13	N/A^d^		
GPs^e^		0 (0)	4	1 (18)		12	1 (17)		14	1 (19)		11	2 (22)		9	6 (75)		8	13.53 (17.72)^f^		
NPs^g^		8 (100)	8	1 (33)		3	4 (67)		6	0 (0)		4	0 (0)		2	1 (0)		—^h^	2.65 (3.04)		
RD^i^		1 (100)	1	2 (100)		2	1 (100)		1	0 (0)		1	0 (0)		1	0 (0)		0	1.19 (1.63)		
Pharmacist		0 (0)	0	0 (0)		1	0 (0)		1	0 (0)		1	0 (0)		1	0 (0)		0	0.63 (0.99)		

^a^Site names were assigned based on the timing of recruitment. Ontario FHT data available from 165 FHT sites.

^b^FHT: Family Health Team.

^c^ePRO: electronic Patient-Reported Outcome.

^d^N/A: not applicable.

^e^eGPs: general practitioners.

^f^Information available from 165 FHTs across Ontario.

^g^NP: nurse practitioner.

^h^Not available.

^i^RD: registered dietitian.

### Patient Recruitment and Eligibility Criteria

Patient recruitment followed exploratory trial procedures, using practice EMRs to identify patients aged ≥60 years with ≥10 visits to the FHT within the previous 12 months. This number of visits has been identified as an indicator of complexity in previous studies [44] and guided the recruitment strategy for the exploratory trial [33]. Age 60 years was chosen as a cut-off over 65 years as the study’s site leads and primary care knowledge user partners identified that many individuals, particularly those living in rural settings, experience complex care needs at an earlier age. EMR-generated patient lists were given to providers to assess whether these individuals met the additional eligibility criteria: (1) perceived willingness to engage in GOC conversations, (2) ability to use a smartphone or tablet in English or have a caregiver who could do this on their behalf, (3) capable of providing consent to participate, (4) willing to complete surveys every 3 months thereafter until the trial concluded. Previous studies have identified that the provider knowledge of the patient is often necessary to identify complexity given the high degree of patient variability [38].

Posters describing the study were hung in waiting rooms at sites, and the study was presented at chronic disease management programs that targeted patients with chronic disease and complex care needs for patient self-identification. Some patients self-identified as eligible after presentations at the programs, but none came to the study via posters. Recruitment materials and processes were built on what was learned from the exploratory trial and were reviewed and modified by the project’s patient partner. Recruitment occurred during a scheduled office visit or by phone by a research coordinator assigned to that site. Patient and provider consent was obtained before randomization.

The minimum sample size required for the recruitment of sites and patients was determined using closed-form analytic formulas with a power of 80% based on a minimal clinically important difference of our core measure of quality of life (the AQoL-4D) of 0.06 [45], an expected SD in assessment of quality of life (AQoL) of 0.22 [46], an expected intraclass correlation coefficient (ICC) of 0.01 (calculated based on total primary care use over a 1-year period among a 10% sample of the Ontario population, which served here as a proxy measure for patient outcomes), and an expected attrition rate of 10% (rated based on previous studies in similar population groups using similar technology [47]). A minimum sample size of 176 patients was calculated, with a target of recruiting 29 patients per site.

### Technology Training

Providers and patients were trained on the tool before switching from the control to the intervention during an onboarding session. Training for providers was done at the clinic level, where groups of providers were presented the technology by a research team member who walked through the technology by setting up goals for a mock patient. Patients were trained one-on-one with a research team member on how to use the mobile device and platform just before their onboarding visit with the provider. Providers and patients were also provided with user manuals [48] and contact information for the research team for technology support.

### Data Collection

Context, process, and outcome data were collected via patient-reported surveys, interviews, ethnographic observations, and chart audits. Survey and chart audit data were collected across all 6 sites, whereas qualitative data were collected at the 3 case sites (sites A, E, and F). In total, 4 of 6 agreed to participate as case sites, and 1 dropped out as a case site because of low patient recruitment.

#### Patient-Reported Surveys

The primary outcome for this study was health-related quality of life measured using the Assessment of Quality of Life–4 Dimensions (AQoL-4D) [49]. The AQoL-4D is a 12-item questionnaire that addresses the activities of daily living, mental health, relationships with others, and physical aspects of a patient’s quality of life. AQoL-4D responses were aggregated to generate a raw, continuous score with higher scores indicating a greater quality of life. The secondary outcome, self-management, was measured using the 13-item Patient Activation Measure (PAM), which measures the extent to which a patient is activated in their own care [50]. PAM is considered a valid and reliable metric of patient self-management for older adults with multimorbidity [50-52]. PAM generates a score from 0 to 100, with higher scores indicating greater activation (patients are better able to manage their care) [53]. Outcome data were collected at baseline and every subsequent 3 months until the end of the trial. For 3 patients in group 2 who were enrolled in the study late, outcome data were collected between October 2018 and June 2019 (Multimedia Appendix 3).

Patient and provider demographic information were collected at baseline. A chart review was conducted posttrial to collect missing data in the patient demographic forms, particularly the number of types of chronic conditions and medications.

#### Interviews

Semistructured interviews were conducted with patients, providers, and managers at case sites at the midpoint (6 months for sites A and E and 4.5 months for site F) and end of the trial. Interviews were conducted by research team members trained in qualitative data collection, with initial interviews conducted in pairs to ensure consistency in the approach. The interview guide was developed to capture the experiences of patients, providers, and managers using the tool or engaged with the trial. Probes were used to delve into implementation factors found to be important to the intervention in the exploratory trial, for example, patient-provider relationships and team environment [33] (Multimedia Appendix 4). Interviews were conducted in person (with one follow-up midpoint interview conducted over the phone), lasted between 20 and 60 minutes, and were audio-recorded and transcribed verbatim.

#### Ethnographic Observation

Ethnographic observations of case sites occurred at multiple points throughout the study, mainly when conducting other activities such as training, patient onboarding, and interviews. At these points, a member of the research team would observe the clinic visits between the patient and provider. Providers were also encouraged to inform the team when patients were coming in for visits so that additional *ad hoc* observations could be conducted; however, no such invitations occurred. Field memos were taken during and immediately after the observation periods. Field note guides helped research staff attend to contexts and processes anticipated to be relevant based on findings from the exploratory trial. Observations were conducted by research coordinators who had graduate training in qualitative health services methods or were provided training by the project lead in the approach. For coordinators, newer to the method observation debriefs and field memo reviews were conducted by the lead to provide ongoing training and skill building.

#### Use Logs

Use logs from the ePRO tool were used to track tool use and the types of goals set by the participants. Goals were categorized into types using qualitative content analysis. Tool use was determined by the number of interactions defined as any log-in or data entered into the system; participants completing one interaction in a given month were considered *active* that month. The total number of active participants was calculated monthly to categorize participants into long-term (using the app for 3 or more months), short-term (using the app for <3 months), or nonuser (participants who did not use the app after initial onboarding) groups. The 3-month cut-off was consistent with previous mobile health (mHealth) clinical trials [54]. Use categories helped to interpret the qualitative and quantitative findings.

### Data Analyses

#### Statistical Analysis

Descriptive statistics were calculated for the cohort stratified by groups of FHTs using counts and mean (SD) values for categorical and continuous variables, respectively. To estimate the degree to which the ePRO tool plus usual care affects health-related quality of life and self-management relative to usual care alone, linear models were fitted with exposure identified by a fixed-effect ordinal variable of calendar time (accounting for staggered implementation) and adjusting for clustering at the FHT site level [55]. The primary effect estimates are summarized as the mean differences for continuous outcomes. Each comparison was evaluated using a 2-sided test at a nominal significance level of α=.05. Statistical analyses of outcome data were performed using the intention-to-treat principle. All descriptive analyses and multilevel modeling were completed using Stata 15.1 statistical software (StataCorp LLC). Owing to the size of the data set, mixed effects models that included covariates such as age, sex, income level, rurality, chronic disease management, and number of chronic conditions could not be included in the modeling.

While missingness in cluster randomized trials in primary care can be handled via multiple imputation methods, using any such imputation to estimate the absence of data points in the cohort was deemed inappropriate owing to the high degree of missingness [56,57]. Aligned with the intention-to-treat approach, individuals were not excluded from the analysis based on their nonresponses to the survey; only variables were excluded, not individuals.

#### Interview and Observation Data

Interview and observational data were used to address the second research question and were analyzed using inductive qualitative descriptive [58] and narrative descriptive methods [59], with separate analyses conducted for patient and provider interviews. Manager interviews were coded with provider data as they were asked similar questions and addressed many of the same implementation constructs. Consistent with this method, codes that described the dominant themes within participant groups were identified. The coding was conducted by researcher pairs trained using qualitative methods to support the validation. Observational memos were coded with patient interviews and were also reviewed as part of the analytic process to provide context information where appropriate to guide interpretation. All team members involved in qualitative data collection and analysis were trained to attend to reflexivity in their approach and all kept fulsome analytic memos to track their own positionality with regard to the qualitative analysis.

To support directed analysis for the purposes of this evaluation, a deductive approach was used to map descriptive codes to Normalization Process Theory (NPT) [60] to understand implementation mechanisms. Exploratory trial findings suggest that NPT is a likely theory of change that underpins this intervention [33]. NPT suggests that new processes become embedded as part of actors’ routines through the social production of work, enabled through 4 generative mechanisms: coherence, cognitive participation, collective action, and reflexive monitoring (Multimedia Appendix 5 offers descriptions of the concepts). These 4 NPT constructs were applied to descriptively coded patient, provider, and manager interviews and observational data, and cross-referenced with patient user groups (long, short, and non) and case site characteristics to generate insights regarding factors that drove implementation and outcomes. Data coded to relevant themes were extracted and organized using tables using a framework analysis approach [61] to identify patterns and trends. The research team reviewed the tables as part of the qualitative validation (supporting credibility and trustworthiness). NVivo 11 software (QSR International, version 11, 2015) was used to manage data in the initial coding phase, and Microsoft Excel and Word files were used to organize data for the framework analysis.

#### Integrating Quantitative and Qualitative Data

Integration of quantitative and qualitative data sets followed a *convergent design* that involves collecting all sources of data concurrently, separately analyzing data, and then comparing results through interpretation and discussion of findings [62,63].

### Ethics

Research ethics approval was granted by the University of Toronto’s Health Sciences Research Ethics Board (#33944) and the ethics committees of all participating practices.

## Results

### Participant Recruitment

**Figure 6:**
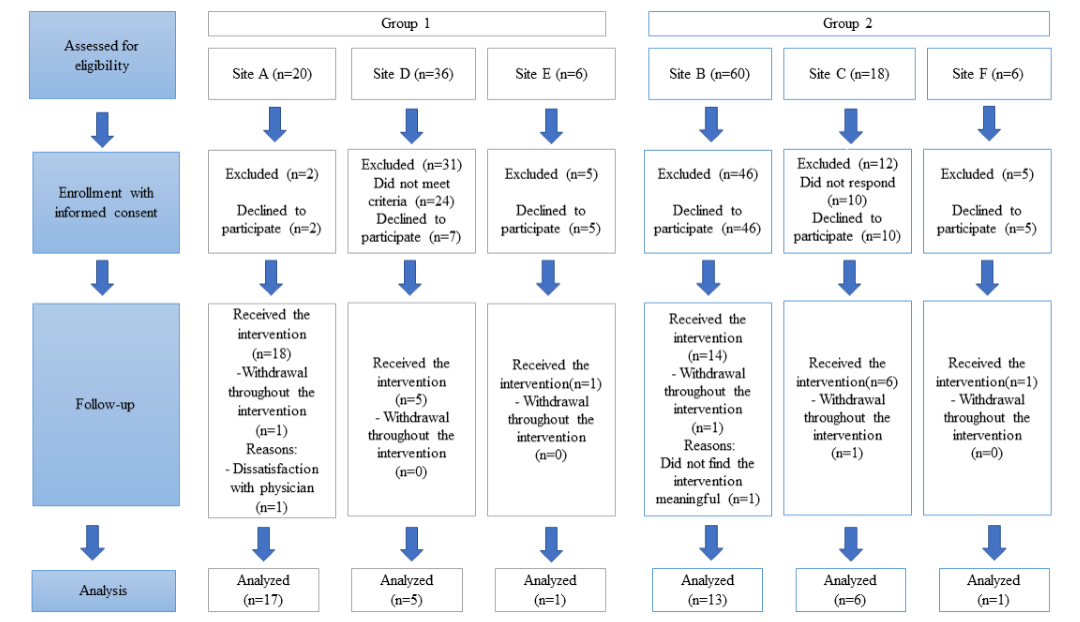
CONSORT (Consolidated Standard of Reporting Trials) flow diagram of patient recruitment arranged by group.

**Figure 7:**
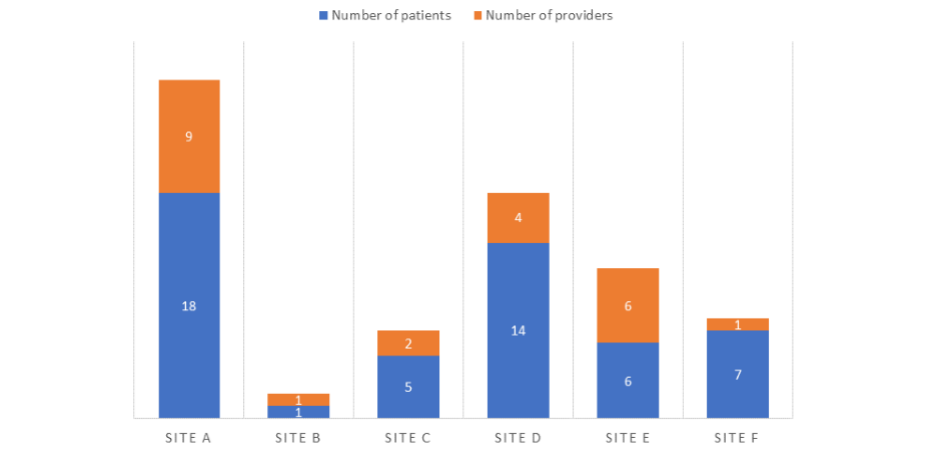
Number of providers and patients participating at each site.

Although the study design target was 176 patients, only 142 (80.6%) were identified as eligible and approached. Of the 142 approached, 46 (32.4%) consented to participate. One participant withdrew before any data collection, leaving 45 (31.6%) participants. This relatively low acceptance rate is an additional challenge. Patient-reported reasons for not participating included perceiving that they did not have complex or chronic conditions, lack of time, perceived conflicts with other life responsibilities (eg, planned vacations and travel), feeling as though they did not have a goal to work on, or were uninterested in this research. In total, 7% (3/45) patients dropped out of the study (1) because of a decline in health condition, making it difficult to participate, and (2) because of loss of interest in participating. Figure 6 shows the CONSORT flow diagram depicting patient recruitment. Figure 7 offers a summary of the number of patient and provider participants per site.

### Participant Characteristics and Goals Set

Patient demographics are summarized in Table 2 and presented by randomized groups to allow for between-group comparisons. There was a statistically significant difference in the rurality and socioeconomic status between the groups.

Patients set a variety of goals related to self-management, mental health, social health, and overall well-being and self-care (Multimedia Appendix 6). Patient-provider pairs varied in terms of the degree of specificity of the goals they set, ranging from highly specific goals (eg, walking 20 minutes 3 times per week or losing 10 pounds) to more general goals (eg, reducing meat consumption or getting more sleep).

**Table 2:** Baseline characteristics of the cohort of Family Health Team patients with complex chronic diseases and disabilities (n=44)^a^.

Characteristics		Group 1 (site A, site D, and site E; n=23)	Group 2 (site B, site C, and site F; n=21)	*P* value	
Age (years), mean (SD)		68.65 (7.10)	71.98 (6.20)	.08	
**Sex, n (%)**					.07
	Female	15 (65.22)	7 (33.33)		
	Male	8 (34.78)	14 (66.67)		
**Place of residence, n (%)**					.08
	Urban	9 (39.13)	14 (66.67)		
	Rural	14 (60.87)	7 (33.33)		
**Living alone, n (%)**					.36
	Yes	10 (43.48)	6 (28.57)		
	No	13 (56.52)	15 (71.43)		
**Born in Canada, n (%)**					.17
	Yes	19 (82.62)	13 (61.90)		
	No	4 (17.39)	8 (38.10)		
**Family income [CAD $ (US $)]^b^, n (%)**					.04
	0-29,000 (0-24,199)	7 (30.43)	1 (4.76)		
	30,000-59,000 (24,200-48,398)	7 (30.43)	5 (23.81)		
	60,000-89,000 (48,399-72,598)	2 (8.70)	8 (38.10)		
	>90,000 (>72,599)	7 (30.43)	7 (33.33)		
**Education^c^, n (%)**					.02
	Less than high school	4 (17.39)	1 (4.76)		
	High school	4 (17.39)	1 (4.76)		
	Some college or university	9 (39.13)	4 (19.05)		
	University (undergraduate or graduate)	6 (26.09)	15 (71.43)		
**Ethnicity, n (%)**					.43
	East Asian	0 (0.00)	1 (4.76)		
	South Asian	1 (4.35)	0 (0.00)		
	Metis	0 (0.00)	1 (4.76)		
	White (North American or European)	21 (91.30)	17 (80.95)		
	Mixed heritage	1 (4.35)	2 (9.52)		
**Chronic disease management program, n (%)**					>.99
	Yes	6 (26.09)	2 (9.52)		
	No	1 (4.40)	1 (5.00)		
	Missing	16 (69.57)	18 (85.71)		
Total number of chronic conditions, mean (SD)		4.21 (2.00)	3.20 (2.00)	<.001	
**Chronic conditions diagnoses, n (%)**					
	Arthritis	7 (30.43)	2 (9.52)	—^d^	
	Asthma	5 (21.74)	3 (14.30)	—	
	Atrial fibrillation	1 (4.40)	2 (9.52)	—	
	Cancer	8 (35.00)	3 (14.30)	—	
	Chronic obstructive pulmonary disease	10 (44.00)	2 (9.52)	—	
	Congestive heart failure	0 (0.00)	0 (0.00)	—	
	Diabetes	10 (44.00)	3 (14.30)	—	
	Enlarged prostate	0 (0.00)	6 (29.00)	—	
	Epilepsy	1 (4.40)	0 (0.00)	—	
	Gastroparesis	1 (4.40)	0 (0.00)	—	
	Hypercholesterolemia	13 (56.52)	4 (19.04)	—	
	Hypertension	15 (65.22)	8 (38.10)	—	
	Hypothyroidism	3 (13.04)	0 (0.00)	—	
	Ischemic heart disease	0 (0.00)	2 (9.52)	—	
	Kidney failure	2 (9.00)	1 (5.00)	—	
	Macular degeneration	1 (4.40)	0 (0.00)	—	
	Mental health conditions	1 (4.40)	0 (0.00)	—	
	Pain	6 (26.10)	6 (29.00)	—	
	Sleep apnea	2 (9.00)	3 (14.30)	—	
	Stroke	4 (17.40)	3 (14.30)	—	
	Urinary retention	0 (0.00)	0 (0.00)	—	
	Other^e^	5 (21.74)	6 (29.00)	—	

^a^Balance in the distribution of covariates between group 1 and 2 family health team sites was assessed using the Kruskal Wallis and Fisher exact test. Percentages may not be equal to 100% because of rounding.

^b^Family income before taxes in CAD $. US $1=CAD $1.3.

^c^University indicates individual has either completed a degree or is currently an undergraduate or graduate student.

^d^Missing data not applicable as there were not enough data per individual chronic illness to generate a meaningful *P* value.

^e^Mood disorders (anxiety or depression), multiple sclerosis, acute myocardial infarction, peripheral vascular disease, peripheral neuropathy, and osteoporosis.

### Intervention Impact on Quality of Life and Self-management

Missing survey data were substantial, ranging between 14% and 91%, mainly because of nonresponse rather than attrition. There were 2 individuals who withdrew during the trial; therefore, the loss to follow-up was 4.5%.

Tables 3 and 4 present the descriptive statistics of the AQoL-4D and PAM scores from the sites at each data collection time point, where the *gray boxes* represent the control periods. Raw AQoL scores over time suggest that most patients (with the exception of those at site B) started and remained relatively healthy over the course of the study (with a notably wide SD).

After adjusting for the covariate of time in the model, patients with ePRO combined with usual care (mean 15.28, SD 18.60) demonstrated a nonsignificant decrease in quality of life (t_24_=−1.20; *P*=.24) as compared with usual care only (mean 21.76, SD 2.17). With regard to patient engagement, ePRO combined with usual care (mean 66.5, SD 17.3) demonstrated a nonsignificant decrease in patient activation, t_27_=−1.41; *P*=.17, as compared with usual care (mean 59.49, SD 9.60).

No patterns were evident when exploring descriptive trends in outcomes related to ePRO user intensity (eg, those who used the tool regularly versus those who rarely used or abandoned it all together). There were fewer completed follow-up surveys in the short term and nonuser groups.

**Table 3:** Mean (SD) of patient health-related quality of life at each discrete time point^a,b^.

Calendar time	Group 1				Group 2		
	Site A (n=17)	Site D (n=5)	Site E (n=1)	Site B (n=13)		Site C (n=7)	Site F (n=1)
Baseline (January 2018)	*19.30 (10.10)^c^*	*22.22 (20.03)*	*6.00*	*10.61 (6.78)*		*28.00 (16.78)*	*17.00*
Period 1: April-July 2018	*20.94 (7.32)*	*28.47 (10.72)*	*6.00*	*11.11 (11.50)*		*31.00 (35.40)*	*6.00*
Period 2: July-October 2018	15.83 (7.64)	28.47 (22.00)	—^d^	*10.00 (5.74)*		*37.04 (23.62)*	*11.11*
Period 3: October 2018-January 2019	18.00 (10.00)	20.83 (25.53)	—	8.00 (8.19)		42.00 (35.40)	17.00
Period 4: January-April 2019	22.83 (12.94)	28.70 (13.13)	—	10.42 (9.00)		8.33 (8.00)^e^	22.22
Period 5: April-July 2019	11.11 (3.00)	36.11	—	—		—	—

^a^AQoL scoring 0 to 45 with 45 being the worst possible health.

^b^Mean (SD) quality-of-life scores could not always be calculated for each site and period because of missingness or lack of variability in the questionnaire responses.

^c^Italicization represents the usual care (control) period of the intervention.

^d^Missing data.

^e^For this site, there were only 2 respondents in periods 3 and 4. The two who responded in period 3 had a wide spread between scores (16.67 and 66.06), and the 2 respondents in period 4 were both lower overall (2.77 and 13.89).

**Table 4:** Mean (SD) of patient self-activation scores at each discrete time point during the trial^a^

Calendar time	Group 1				Group 2		
	Site A (n=17)	Site D (n=5)	Site E (n=1)	Site B (n=13)		Site C (n=7)	Site F (n=1)
Baseline (January 2018)	*60.42 (15.00)* ^b^	*61.10 (9.00)*	*53.20*	*63.10 (15.00)*		*55.00 (11.30)*	*58.10*
Period 1: April-July 2018	*60.01 (10.59)*	*58.80 (8.26)*	*56.00*	*72.00 (19.12)*		*52.10 (1.60)*	*66.00*
Period 2: July-October 2018	70.00 (14.92)	63.10 (10.00)	56.00	*69.40 (19.04)*		*59.20 (9.00)*	*58.10*
Period 3: October 2018-January 2019	71.79 (20.31)	53.00 (15.00)	—^c^	68.00 (25.82)		56.30 (13.10)	56.00
Period 4: January-April 2019	68.57 (19.41)	63.00 (4.20)	—	98.00 (5.00)		56.00 (7.00)	61.00
Period 5: April-July 2019	73.57 (13.94)	48.90	—	76.93 (20.54)		—	66.00

^a^Mean (SD) patient self-activation scores could not always be calculated for each site and period because of missingness or lack of variability in the questionnaire responses.

^b^Italicization represents the usual care (control) period of the intervention.

^c^Missing data.

### Mechanisms Likely Driving Outcomes: Selected Findings From Ethnographic Case Studies

Use log data revealed significant attrition on the tool for both long-and short-term user groups with 46% (21/46) of patients using the tool as intended, 15% (7/46) discontinued use after 3 months, and 36% (17/46) abandoned the app after initial training. Data from the ethnographic case studies are analyzed to provide insights into factors that may drive use and potentially influence outcomes.

Table 5 presents a summary of the data sources, and Multimedia Appendix 5 offers a summary of NPT constructs and analysis.

**Table 5:** Ethnographic data sources.

Case sites	Patient interviews (n=24)	Provider interviews (n=22)	Observations (n=21)
Site A	6 midterm 5 end of project	6 midterm 5 end of project	1 onboarding 5 ad hoc
Site B	3 midterm 3 end of project	4 midterm 3 end of project	1 onboarding 9 ad hoc
Site C	2 midterm 2 end of project	2 midterm 2 end of project	2 onboarding 3 ad hoc

#### The Role of Coherence, Cognitive Participation, Collective Action, and Reflexive Monitoring

**Figure 8:**
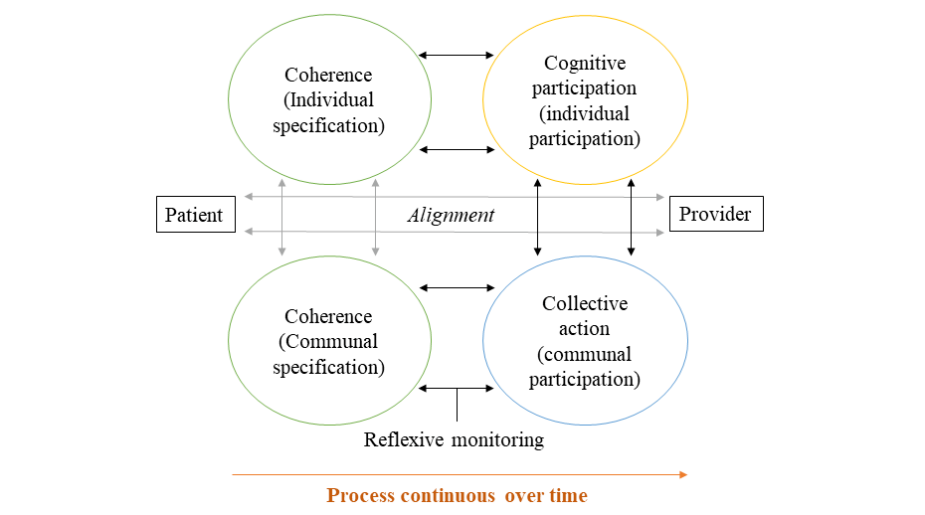
Visual depiction of the normalization process of the electronic Patient-Reported Outcome tool.

Consistent with findings from the exploratory trial, the meaningfulness of the ePRO tool to patients and providers had a significant influence on how and when it was used. Revealed in this analysis, is that the meaningfulness of the ePRO tool (its coherence to the participants) changed over time, and was reliant on (1) alignment to previously held notions of chronic disease management by providers and patients; (2) alignment to daily work and life activities (enabling cognitive participation); (3) strong relationships between patients and providers (enabling collective action); and (4) consistent positive assessments of the tool’s utility (regular reflexive monitoring). An additional challenge is the interactional aspect of the ePRO tool, which means that both individual and collective coherence need to be aligned to the tool, as depicted in Figure 8.

The figure offers a simplified illustration of a complex ongoing process, highlighting 2 key drivers of adoption in this study. The first is the need for alignment between how individuals within a shared process understand that process (coherence) and then act on it (cognitive participation and collective action), depicted by gray arrows in the figure. Collective action (the actual use of the tool) proved to be highly influenced by this individual and shared coherence but differed depending on where users were in the process of GOC. For example, the data demonstrate that collective action occurred more toward the beginning of the intervention during goal-setting, when there was better alignment between individual and communal coherence of the intervention. This important time variable is indicated by the orange arrows. Second, the evaluation and assessment of the tool (reflexive monitoring) is continuous and interactive rather than a demonstration of normalization, as originally theorized, depicted in Figure 8 as black bidirectional arrows. As participants moved through participation and action in using ePRO, they consistently reflected on their individual and collective coherence, assessing whether it was worth continuing. Our data suggest that when alignment is high between individual- and group-level coherence, there is a greater likelihood of ongoing collective action; in this case, the use of the ePRO tool. This relationship is not currently depicted in the tool, as it will need to be tested in future studies.

## Discussion

### Participants and Study Implementation

Only 142 eligible patients of the total 176 patients were identified. The minimum recruitment numbers could not be reached owing to 3 challenges. First, some sites had few provider participants join the study. The usability study and exploratory trial suggested that providers who were just starting with the ePRO tool should manage a maximum of 5 patients at a time to reduce burden. The recruitment strategy required 6 to 8 providers to identify 5 to 10 patients each whom they could manage for the duration of the trial. As such, sites with fewer participants identified fewer patients to participate in the study (sites B and C in particular). Second, the requirement that patients *be ready* to engage in goal-setting proved to be a more significant barrier than at previous stages of ePRO testing. Practice EMRs identified many potential patient participants, but when reviewed by providers, few were identified as *ready.* Related to this point, the stepped-wedge design requires all participants to start an intervention at the same time. This rigid timing created an unanticipated third challenge.

The patient participants also likely represent a *healthier* group overall. Compared with similar patients in Canada, the United States, Australia, and the United Kingdom, patient participants had a lower number of reported chronic illnesses and a higher level of reported education [64-67]. The AQoL scores of patients were aligned with previously published population norms [68]. Participants PAM standardized scores demonstrate slightly higher activation levels as compared with similar multimorbid populations, for instance, in a validation study of PAM that found a mean score of 56.6 (SD 12.9) [51].

Another important point to highlight is the relatively large number of registered dietitians and nurse practitioners who participated. One systematic review found several examples of dietitian-supported diabetes prevention programs [69], and nurse practitioners have been shown to successfully support digitally enabled chronic disease management programs in outpatient settings [70]. These examples, along with findings from this study, suggest an important role for allied health professionals in the implementation of digitally enabled health interventions for chronic disease populations in primary care settings.

Finally, the nature of the research process itself influenced trial implementation and outcome, as it conflicted with the natural process of delivering GOC. First, providers were exasperated by recruitment challenges, which resulted in delays in the trial start date. Second, while having providers manage few patients’ reduced burden, it also meant providers had fewer opportunities to engage with the tool. As time went on, providers began to forget about the tool and why they valued it in the first place. Finally, the stepped-wedge required a time-bounded window for patient onboard. GOC, however, is a fluid approach in which goal-setting needs to occur at a point when patients are ready (as noted in the provider data around coherence). Providers expressed frustration that study parameters limited their ability to onboard patients later identified as individuals who could benefit from the tool.

### Principal Findings

Recruitment challenges previously described resulted in the study being underpowered. As such, a conclusion regarding the effectiveness of the ePRO tool cannot be drawn. Analysis of the ethnographic data reveals interrelationships between use patterns, outcome trends, and patient and provider contexts to reveal the underlying mechanisms driving this complex intervention. Many patients and providers perceived the ePRO tool as valuable with the potential to improve engagement and healthy behaviors; however, over time, this excitement waned. Providers reverted to old ways of working, as did some patients. Waning engagement is not unique to digital health and has occurred in other behavior change interventions. Other patients for whom the tool was well-aligned to their values and aimed to manage their health demonstrated long-term adherence. For those *high users* whose coherence of the tool was tied to their interaction and relationship with their provider, they too began to fall away from the intervention as providers became less involved.

These findings uncover 2 tensions that have implications for digital health interventions for patients with complex care needs and multimorbidity in a primary care setting.

### Challenge 1: Supporting Engagement in the Intervention Over Time

Engagement with an intervention is a well-documented challenge in primary care. Studies of medication adherence show similar ranges of adherence (40%-60%) [71,72] for chronic disease populations in primary care settings (ePRO adherence was 44%). *Nonadherence* reduces exposure and can lessen the effect of the intervention [73,74]. However, this lens suggests that it is the patient’s fault for not doing as they are told, rather than placing a critical lens on the intervention itself. Perhaps a more useful lens is to consider engagement both “(1) the extent (eg, amount, frequency, duration, and depth) of use and (2) a subjective experience characterized by attention, interest, and affect” [75].

The ePRO tool experienced low retention rates typical of many mHealth interventions [76], which are connected to both use and subjective experience. The ethnographic findings suggest that subjective experience is linked to patient coherence and the meaningfulness of the tool. This finding is consistent with other studies that have shown that psychological factors such as motivation, expectations of the app, mental health, cognitive burden, and personal relevance will influence patient engagement [75]. Usability of the technology and tech savviness of users can often act as a barrier to ongoing use [77]. The usability analysis for this trial was too extensive to be included in this study. One key finding from the usability analysis presented in another paper, is that tech savviness and usability issues were moderated by the patient-provider relationship, in that patients with stronger regular connections to their providers were more likely to troubleshoot and work through technology challenges regardless of reported *savviness* [78].

Importantly, in this trial, app burnout occurred for patients *and* providers, for whom attrition was similarly linked to reduced use and subjective experience. Primary care providers have also demonstrated declining engagement with interventions over time, an issue identified in the literature as clinical inertia [79]. With continuous interventions, such as GOC, the ePRO study findings suggest that tapping into coherence consistently may improve engagement by both patients and providers.

### Challenge 2: Meaningfulness for the Individual Versus the Group

Alignment of the ePRO tool to what was important and meaningful to patients and providers (coherence) was foundational. The study findings not only lend support for the importance of meaningfulness in technology [80] but also demonstrate how meaningfulness is constructed at both the individual and group levels, as suggested by NPT [60]. The disconnect between how providers and patients approached GOC is likely a contributor to the abandonment of the tool by those patients who sat somewhere between the strongly self-motivated super users and somewhat indifferent nonusers. For patients, GOC was a way to motivate and feel accountable for goals co-constructed with their providers. For many providers, however, GOC was an approach to support patient self-management, which did not require the same amount of ongoing connection and feedback expected from patients. This view is well-represented by the quote from the physical therapist at site A, as shown in Multimedia Appendix 5, under the cognitive participation domain.

This approach to self-management suggests that a provider plays the role of a consultant, guiding patients through the management of their illnesses [81]. Although there is room for collaborative care and goal-setting in this model, the emphasis is on setting patients up to succeed and then sending them off. The qualitative data from this study suggest patients wanted more of a *coaching* approach with more touch points and interactions to maintain momentum, particularly for patients who started strong but then fizzled out. Theories of volition and self-regulation suggest that “feedback focused on the immediate benefits of behavior may be optimal during the early stages of behavior change” but can be reduced as individuals become more intrinsically motivated and confident [82]. What perhaps happened here is those patients who fizzled out were still at their *early stage* and, as such, required more engagement to keep moving forward. This finding suggests the need to better calibrate coherence when implementing digital health solutions with diverse user groups over time. Future studies should also probe how variation in the degree of goal specification found in this study may also influence patients at different stages of behavior change.

### Strengths, Limitations, and Future Research

Similar to comparable studies of digital health interventions in primary care [83], both site and patient recruitment challenges were experienced. In addition, some values in the sample size calculation, such as attrition, were underestimated. Underpowering meant that all confounding variables collected via demographic baseline surveys and chart reviews could not be included in the modeling. In addition, some context data (notably participation in chronic disease management programs) may have changed over time and were only collected once at baseline. The smaller than anticipated sample size did allow for a more robust approach to ethnographic data collection, resulting in a rich, qualitative data set, which is a strength of the study. Future studies in primary care settings should consider both the setting context and the nature of the intervention being tested to better align trial methods to real-world implementation. More flexible adaptive trials or the application of an interrupted time series within the clusters may be more appropriate in these dynamic environments. Further exploration as to the reason why some providers were more successful in identifying eligible patients as compared with others in the study is another potential area of study to better understand this implementation challenge.

The findings may not be widely generalizable to older adults with complex needs, as patients in this study were generally healthier and more educated. However, the high proportion of complex older women living in rural environments in this project addresses a notable gap in the evidence on interventions for this population [84,85]. Relying on provider screening may have led to selection bias, which can reduce generalizability. However, as there is a lack of consensus on the definition of patient complexity, reliance on physician expertise and self-identification has been found to be a viable approach to identify this patient population [86].

Another important limitation is that this study lacks additional data on provider characteristics, such as age, years of experience, and employment status (eg, full-time or part-time). While a baseline demographic survey was deployed to all providers, few remitted these surveys despite multiple attempts to collect the data either via email, phone, or in person. While some key variables, such as comfort with technology, were collected via interviews, the other demographic variables would have aided in interpreting data and supported generalizability to other similar provider groups.

While this study offers a multimethod view of the effectiveness of the ePRO tool, the findings presented here focus on the major themes that emerged in the analysis. Further analyses will explore the interrelationships between NPT constructs and other context variables, in particular how these concepts relate to professional identities, organizational culture, and notions of how best to engage in chronic disease management. An important lesson from this trial is how the nature of GOC and chronic disease management in primary care settings is a fluid and complex process that is often unique to particular settings and provider-patient pairs. Highly adaptive trial designs, which allow the study to align to these contexts more closely, may have greater success in engagement for longer interventions.

### Conclusions

Although this study is unable to provide a definitive answer to the effectiveness of the ePRO tool, it did generate novel insights regarding the implementation of digital health technologies in primary care settings. The findings demonstrate the critical role of coherence, or meaningfulness, of an intervention, and the great challenge of aligning coherence across diverse user groups over time. Future work in this area should pay careful attention to how chronic disease management, GOC, and self-management are understood and pursued when implementing digital health technologies to advance these models of care.

## Appendix

Multimedia Appendix 1 PRECIS-2 (Pragmatic Explanatory Continuum Indicator Summary) Wheel domain description.

Multimedia Appendix 2 Electronic Patient-Reported Outcome wireframes.

Multimedia Appendix 3 Data collection schedule.

Multimedia Appendix 4 Sample interview guide questions.

Multimedia Appendix 5 Summary of how patients and providers understood, engaged with, and reflected on the adoption of the electronic Patient-Reported Outcome tool as aligned with Normalization Process Theory constructs.

Multimedia Appendix 6 Types of goals and tasks set during the trial.

## Abbreviations

AFHTO: Association of Family Health Teams of Ontario
AQoL-4D: Assessment of Quality of Life–4 Dimensions
CIHR: Canadian Institutes of Health Research
eHIPP: eHealth Innovation Partnership Program
EMR: electronic medical record
ePRO: electronic Patient-Reported Outcome
FHT: Family Health Team
GOC: goal-oriented care
GP: general practitioner
mHealth: mobile health
NP: nurse practitioner
NPT: Normalization Process Theory
PAM-13: Patient Activation Measure–13 item
RD: registered dietitian
RN: registered nurse
SMART: specific, measurable, attainable, realistic, timely
