# Supplementary material for: Assessing the Implementation and Effectiveness of the Electronic Patient-Reported Outcome Tool for Older Adults With Complex Care Needs: Mixed Methods Study
Source: J Med Internet Res. 2021 Dec 2;23(12):e29071. doi: 10.2196/29071 (PMC8726765; doi:10.2196/29071)
Supplement: Multimedia Appendix 1 [file jmir_v23i12e29071_app1.docx]

# Mulitmedia Appendix 1: PRECIS-2 Wheel domain description

|  | **Domain** | **Score** | **Rationale** |
| --- | --- | --- | --- |
| 1 | Eligibility Criteria | 5 | The trial had broad inclusion criteria, few exclusion criteria, and recruited participants from multiple centers. Patients were also able to self-identify as eligible. |
| 2 | Recruitment Path | 5 | Participants were recruited during a usual clinic visit or chronic disease management program. We did not recruit participants through social media or public advertisement. |
| 3 | Setting | 5 | The trial took place in six primary care setting across Ontario. The characteristics of the primary care settings where the trial took place was identical of targeted patients’ usual care settings. |
| 4 | Organization intervention | 4 | To use the ePRO app effectively and set goals, primary care providers were trained in using the app. Patients were also provided training as well as mobile phones for the duration of the trial, however, had the option to use their own. Apart from that technology training, ePRO implementation in the primary care model did not require any more than usual resources, skills/ expertise or change in care delivery model for the providers or organization. |
| 5 | Flexibility of experimental intervention-Delivery | 5 | Typically, the trial participants received one training session and then left to the individual participants’ decision, who could decide their frequency and nature of app usage. Patients remained under the care of their own care team throughout the trial. The trial did not require for patients to alter their frequency and nature of primary care visits. Additionally, providers did not have to alter the way they deliver care to their patients. |
| 6 | Flexibility of experimental intervention-Adherence | 4 | There was no strict monitoring of participants’ behavior/app usage. However, patients were encouraged and reminded about using the app every 3 months when outcome surveys were collected which was a gentle compliance-improvement strategy. However, compliance data were not fed back to the study participants and so compliance data did not inform the participants’ app usage or participation in the study. This was a fairly pragmatic approach. |
| 7 | Follow-up | 3 | There was a minimal follow-up from the research team; however, an extensive amount of data was collected during the intervention period which includes surveys at multiple time points and mid-point interviews. |
| 8 | Outcome | 4 | Patient Activation Measure and Quality of Life measure are patient-important outcomes that measure how patient functions and feel about their well-being. While these general outcomes were identified as important to patients in the development and exploratory trials for this tool, a few patients found the tools did not resonate with their experience in this trial. No physiological measure or surrogate measure was collected from the study participants. |
| 9 | Analysis | 5 | Data were analyzed using the intention to treat protocol with all available data. No participant data were excluded due to non-compliance to the study protocol. |
