# Supplementary material for: Assessing the Implementation and Effectiveness of the Electronic Patient-Reported Outcome Tool for Older Adults With Complex Care Needs: Mixed Methods Study
Source: J Med Internet Res. 2021 Dec 2;23(12):e29071. doi: 10.2196/29071 (PMC8726765; doi:10.2196/29071)
Supplement: Multimedia Appendix 2 [file jmir_v23i12e29071_app2.pdf]

Project:	Bridgepoint ePros	Layout	Portal: Landing Page

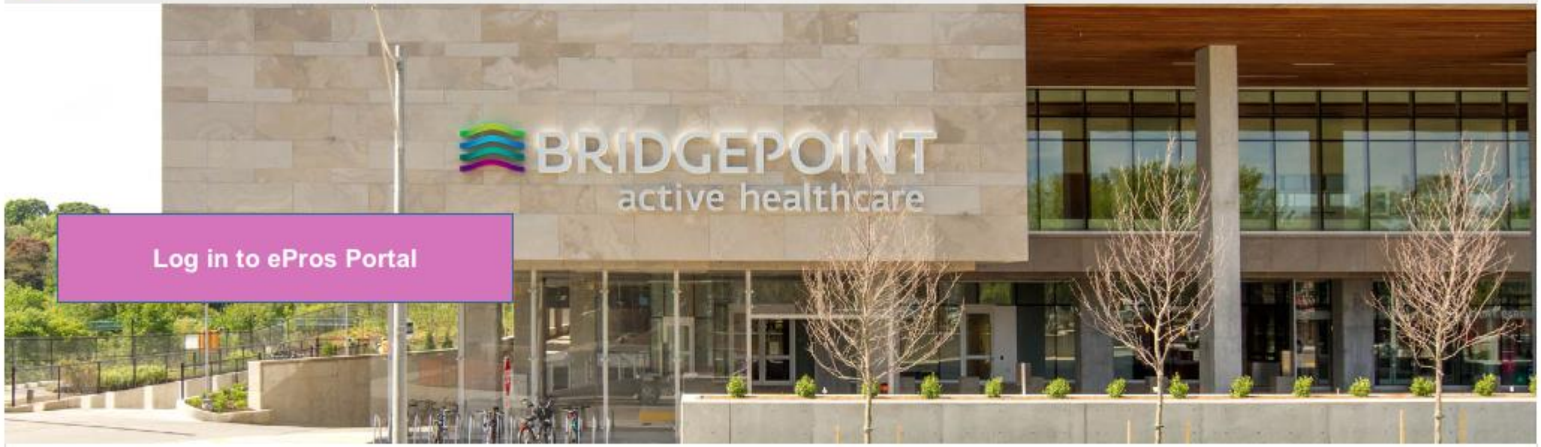

Log in to ePros Portal

Privacy	This Document Is Confidential

Project:	Bridgepoint ePros	Layout	Portal, Provider View: Dashboard

Bridgepoint ePros

User Name

My Profile
Logout

Patient Search Enter Patient Name

Search Results
Patient One
Patient Two

Search results dynamically generated as user types in patient name.

Potential to create "roll-up" attainment metric. To be discussed.

Patients You're Linked To:

Action		Patient Name	Provider Name	Goal Statement	Attainment Trending
Edit	Results	Patient One	Provider One	Maintain Independance	+2
Edit	Results	Patient Two	Provider Two	Greater Mobility	0
Edit	Results	Patient Three	Provider Three	Statement Three	-1

On mouse over, preview detail about Goal Statement will be presented.

Activity Detail

Goals	Act	Attainment Scale
Goal One	80%	+1
Goal Two	90%	+2
Goal Three	10%	-1

Privacy	This Document Is Confidential

Project:	Bridgepoint ePros	Layout	Portal, Provider View: Patient Goal Statement Index

Bridgepoint ePros

User Name

Patient: Patient Name
Provider: Provider Name

Goal Statements | Statement Editor | Outcome Measures

- Add New Goal Statement
- View History

Patient Goal Statements

Action	Goal Statement	Goals	Attainment Trending
Edit	Maintain Independance	3	+2
Edit	Goal Statement Two	2	0
Edit	Goal Statement Three	3	-1

- Edit Goal Statement
- Edit Outcome Measures

When edit option is selected, drop down provides editing options.

On mouse over, preview detail about Goal Statement will be presented.

Activity Detail

Goals	Act	Attainment Scale
Goal One	80%	+1
Goal Two	90%	+2
Goal Three	10%	-1

Privacy	This Document Is Confidential

Project:	Bridgepoint ePros	Layout	Portal, Provider View: Edit Goal Statement, Edit Goal

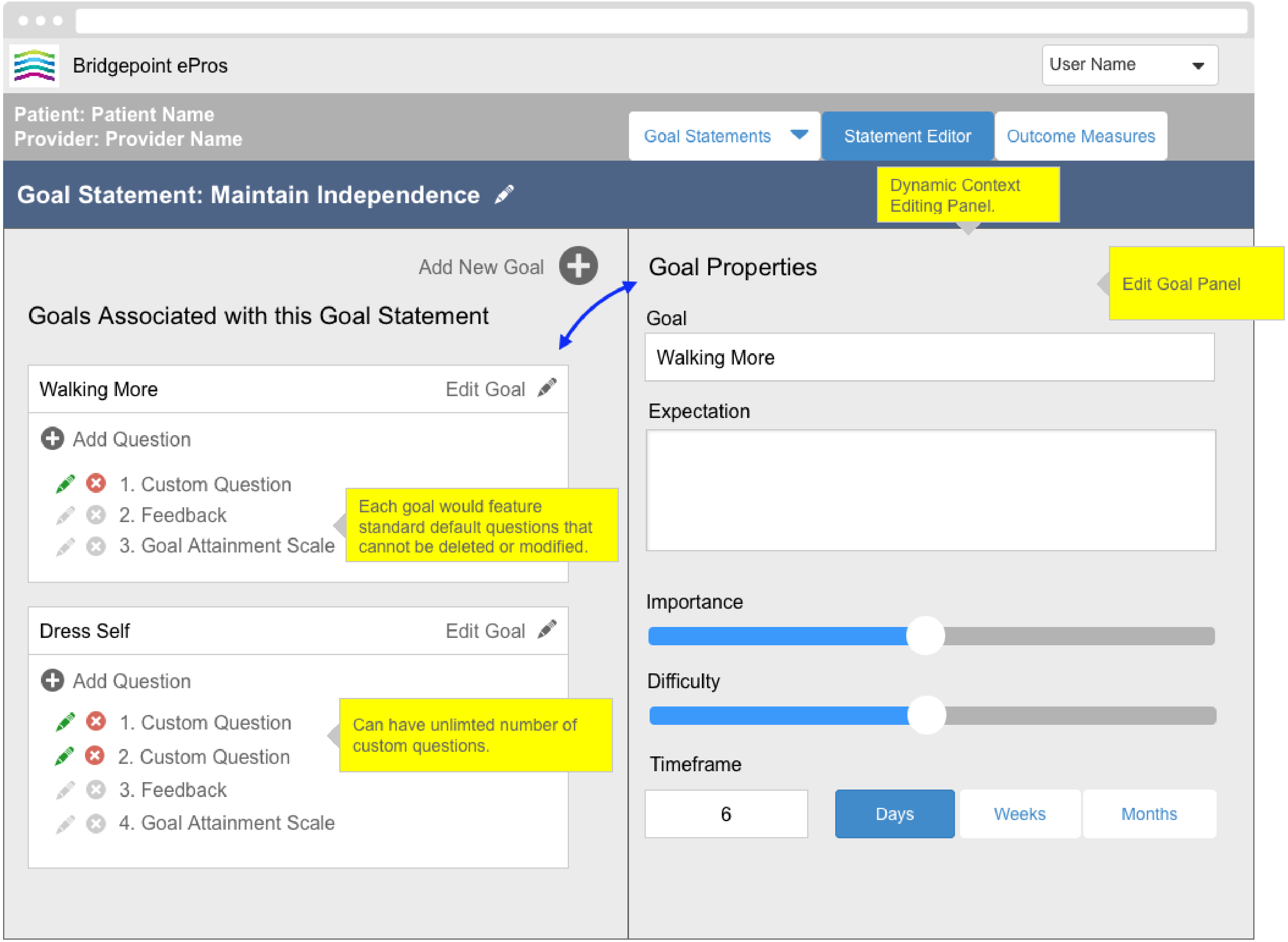

Privacy	This Document Is Confidential

Project:	Bridgepoint ePros	Layout	Portal, Provider View: Edit Goal Statement, Edit Question A

Bridgepoint ePros

User Name

Patient: Patient Name
Provider: Provider Name

Goal Statements | Statement Editor | Outcome Measures

Goal Statement: Maintain Independence

Dynamic Context Editing Panel.

Add New Goal

Goals Associated with this Goal Statement

Walking More — Edit Goal

Add Question

1. Custom Question
2. Feedback
3. Goal Attainment Scale

Each goal would feature standard default questions that cannot be deleted or modified.

Dress Self — Edit Goal

Add Question

1. Custom Question
2. Custom Question
3. Feedback
4. Goal Attainment Scale

Can have unlimted number of custom questions.

Question Properties

Edit Question Panel

Choose Question from Library

Questions You Have Created in the Past — Search

Search box filters questions based on text entered.

Question	Type
Custom Question One	Text, Single Line
Custom Question Two	Multiple Choice
Custom Question Three	Multiple Choice
Custom Question Four	[illegible]

On mouse over question, preview will be displayed.

Preview

How anxious (worried, nervous) do you feel?

1. Not at all anxious
2. A little anxious
3. Moderately anxious
4. Very anxious
5. Extremely anxious

Choose

Predefined Question Library

Question	Type
Library Question One	[illegible]
Library Question Two	[illegible]
Library Question Three	[illegible]
Library Question Four	Analog Scale

Questions Shared by Other Providers — Search

Question	Type
Shared Question One	Text, Single Line
Shared Question Two	Multiple Choice
Shared Question Three	Multiple Choice
Shared Question Four	Analog Scale

Privacy	This Document Is Confidential

Project:	Bridgepoint ePros	Layout	Portal, Provider View: Edit Goal Statement, Outcome Measures

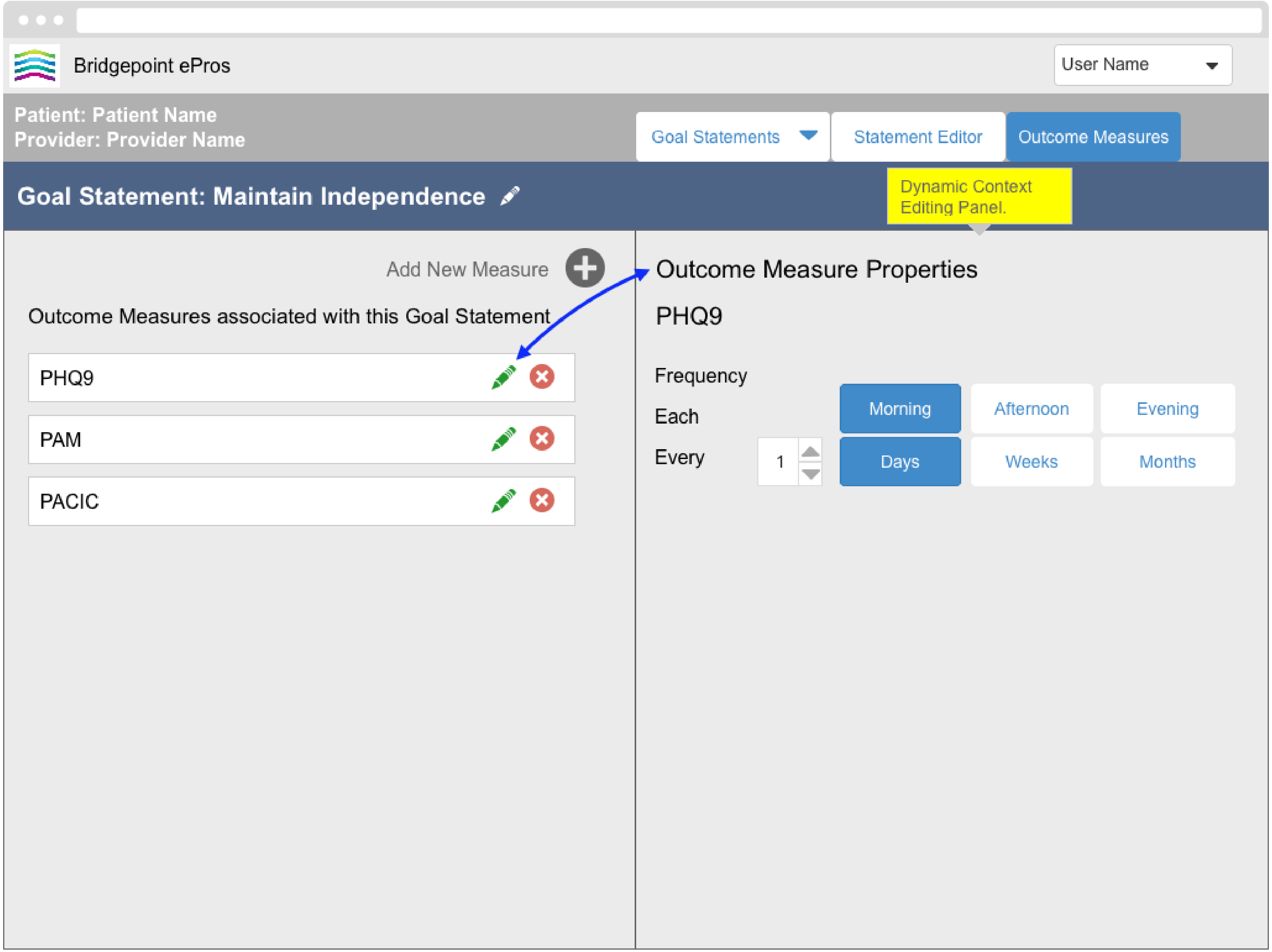

Privacy	This Document Is Confidential

Project:	Bridgepoint ePros	Layout	Portal, Provider View: Patient Goal Statement Index

Bridgepoint ePros

User Name

Patient: Patient Name
Provider: Provider Name

Goal Statement: Maintain Independence

Goal: Walking More

Custom Question One

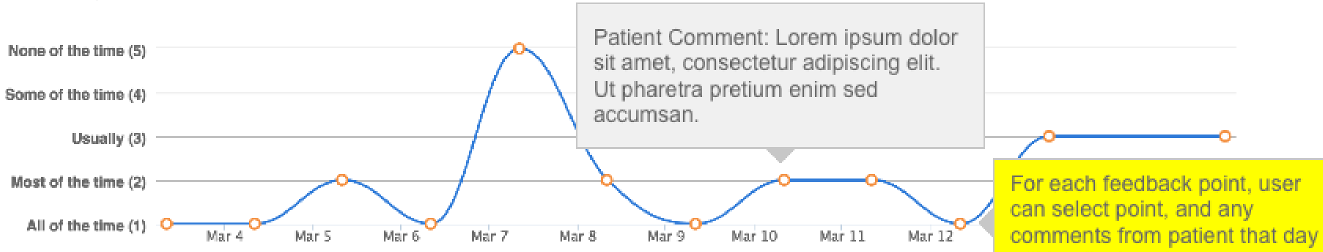

For each feedback point, user can select point, and any comments from patient that day will be displayed.

Goal Attainment Scale

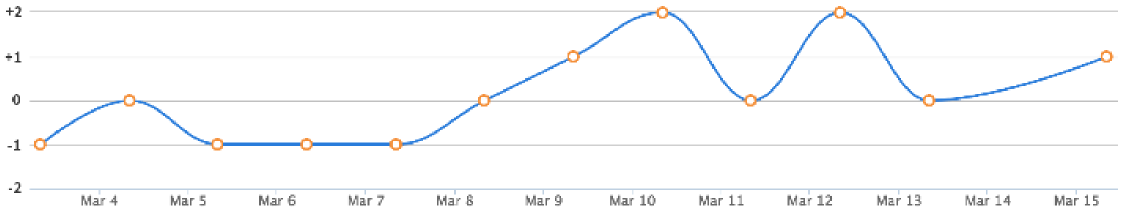

Patient Comments

Patient comments will also be listed by date as part of the results.

Privacy	This Document Is Confidential

Project:	Bridgepoint ePros
Layout	Android Splash Screen

BRIDGEPOINT
active healthcare

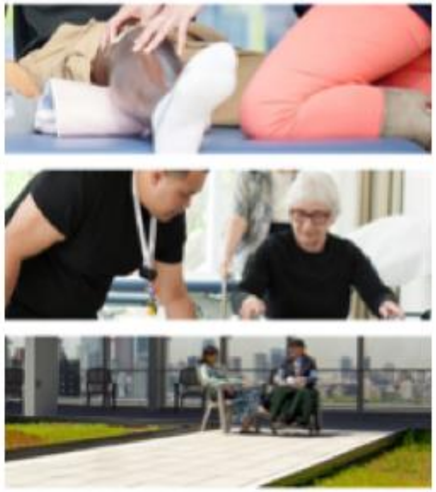

Privacy	This Document Is Confidential

Project:	Bridgepoint ePros
Layout	Home Dashboard

12:00

My Goals

Maintain Independence 4

Lorem ipsum dolor sit amet, consectetur adipiscing elit. Ut pharetra pretium enim sed accumsan. Curabitur tristique, lacus at viverra accumsan, lacus lectus bibendum nibh, nec ultricies risus eros eget nibh.

Feedback reminder, tap here to complete your feedback for this goal.

Other Goal

Lorem ipsum dolor sit amet, consectetur adipiscing elit. Ut pharetra pretium enim sed accumsan. Curabitur tristique, lacus at viverra accumsan, lacus lectus bibendum nibh, nec ultricies risus eros eget nibh.

Privacy	This Document Is Confidential

Project:	Bridgepoint ePros
Layout	Feedback Home

12:00

Maintain Independence

Feedback | Charts | Photos

Today 4

There are 12 active questions for:
Goal: Walk More
Goal: Dress Self

So far you have answered 8 of the required questions. There are also 3 optional questions.

Yesterday ✓

There were 4 active questions for:
Goal: Walk More

So far you have answered 4 of the required questions.

Privacy	This Document Is Confidential

Project:	Bridgepoint ePros
Layout	Feedback Survey A

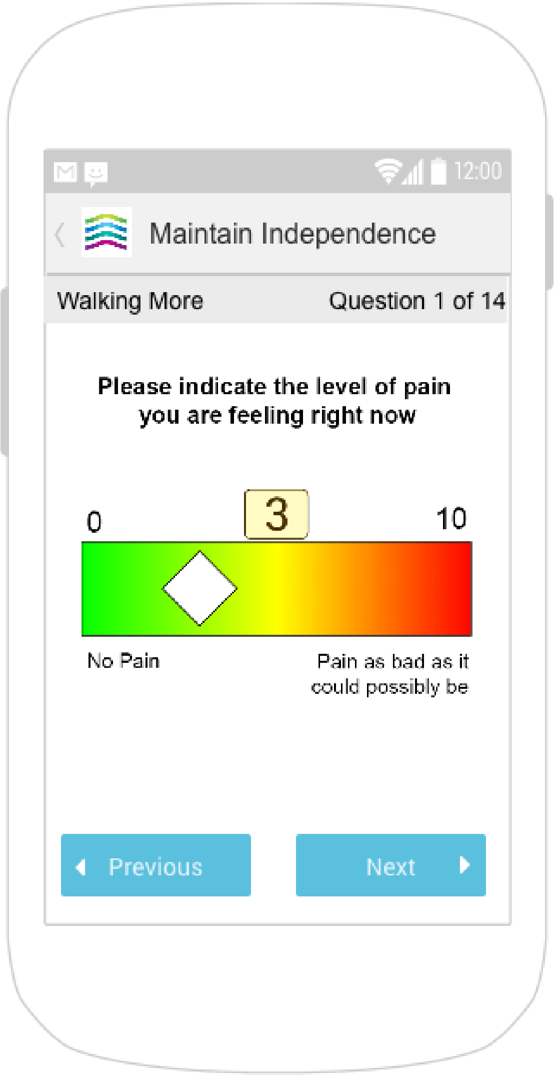

Privacy	This Document Is Confidential

Project:	Bridgepoint ePros
Layout	Feedback Survey B

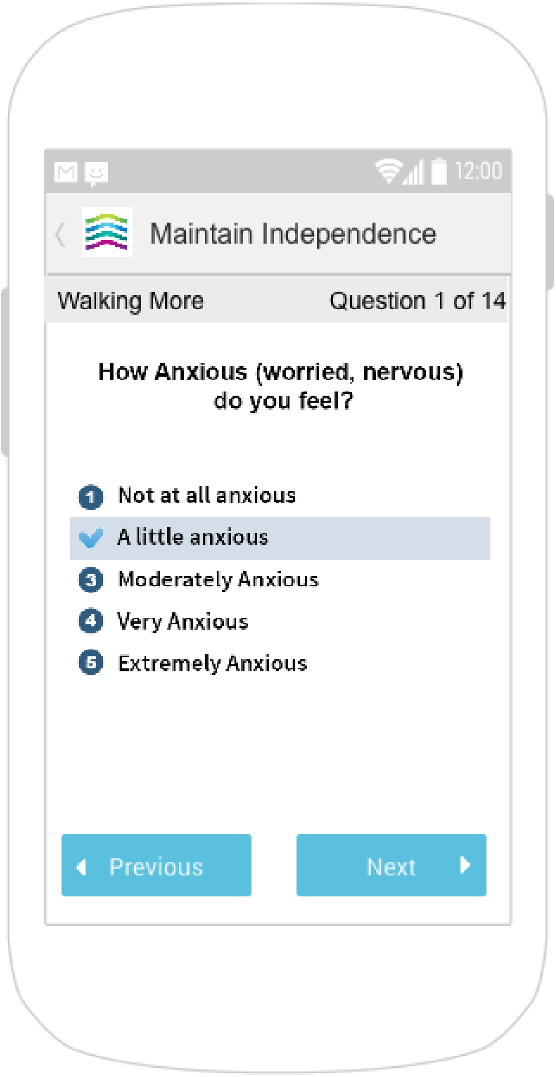

Privacy	This Document Is Confidential

Project:	Bridgepoint ePros
Layout	Charts / Results / Graphs

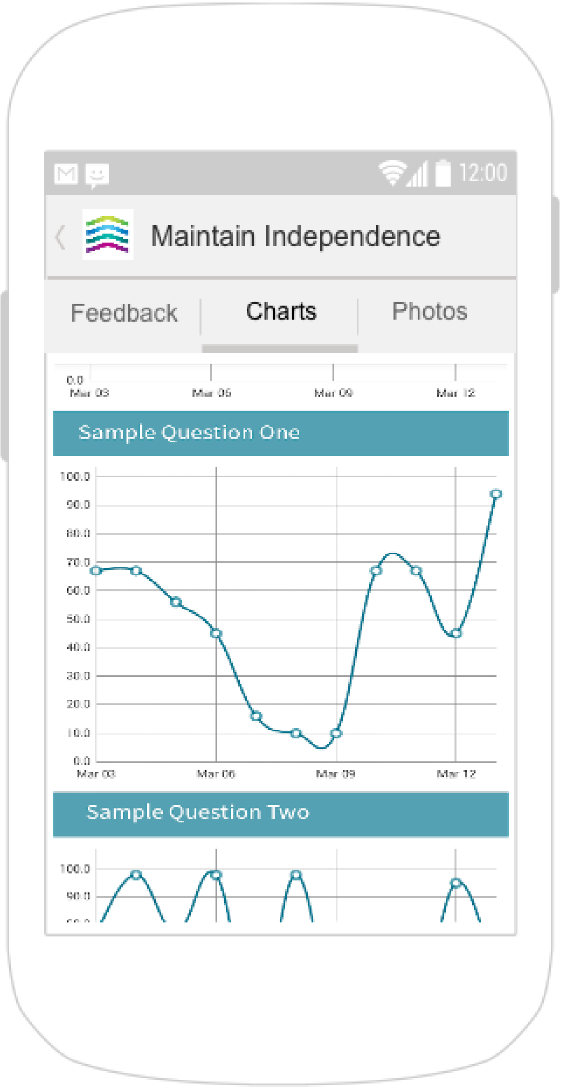

Privacy	This Document Is Confidential
