# Supplementary material for: Assessing the Implementation and Effectiveness of the Electronic Patient-Reported Outcome Tool for Older Adults With Complex Care Needs: Mixed Methods Study
Source: J Med Internet Res. 2021 Dec 2;23(12):e29071. doi: 10.2196/29071 (PMC8726765; doi:10.2196/29071)
Supplement: Multimedia Appendix 3 [file jmir_v23i12e29071_app3.docx]

# Multimedia Appendix 3: Data collection schedule

| **Concept** | **Measurement level** | **Variable** | **Tool/method** | **Group 1 (early)**  **Data collection** | **Group 2 (late)**  **Data collection** |
| --- | --- | --- | --- | --- | --- |
| ***Outcome***  Intervention and control sites | Patient | Quality of Life | Assessment of Quality of Life -4D (AQoL-4D) | Baseline  3, 6, 9, 12, 15 months | Baseline  3, 6, 9, 12, 15 months |
|  |  | Self-management | Patient Activation Measure (PAM). | Baseline  3, 6, 9, 12, 15 months | Baseline  3, 6, 9, 12, 15 months |
|  |  | Patient experience | Patient experience survey (from AFHTO and HQO) | Baseline  3, 6, 9, 12, 15 months | Baseline  3, 6, 9, 12, 15 months |
|  |  | Goal-attainment captured by ePRO tool – *intervention sites only* | Goal attainment scaling.  *Completed as part of the intervention.* | Over 12 month intervention period (analyzed monthly) | Over 9 month intervention period (analyzed monthly) |
|  | | | |  |  |
| ***Process***  Intervention sites | Patient | Tool experience | Post-Study System Usability Questionnaire (PSSUQ). | Every 3 months during 12 month intervention | Every 3 months during 9 month intervention |
|  |  |  | Patient interviews | 6 months into intervention and  post-intervention | 4.5 months into intervention and  post-intervention |
|  | Provider | Tool experience | Post-Study System Usability Questionnaire (PSSUQ). | 6 months into intervention and  post-intervention | 4.5 months into intervention and  post-intervention |
|  |  |  | Provider interviews | 6 months into intervention and  post-intervention | 4.5 months into intervention and  post-intervention |
|  |  | Delivering patient-centred care | Provider interviews – drawing on Assessment of Chronic Illness Care (ACIC) tool | 6 months into intervention and  post-intervention | 4.5 months into intervention and  post-intervention |
|  | Organization | Provider workflows | Provider interviews | 6 months into intervention and  post-intervention | 4.5 months into intervention and  post-intervention |
|  | | | | |  |
| ***Context***  Intervention and control sites | Patient | Demographic characteristics | EMR extraction  Patient information sheet | Baseline and post-study | Baseline and post-study |
|  | Provider | Demographic characteristics | Provider information sheet | Baseline | Baseline |
|  | Organization | Size; description of the organization; Resources; Support  Training | Document analysis  Provider and leaders interviews | 6 months into intervention and  post-intervention | 4.5 months into intervention and  post-intervention |
|  | System | Structure  Standardization of data systems  Legal requirements  Funding | Document analysis  Provider and leaders interviews | 6 months into intervention and  post-intervention | 4.5 months into intervention and  post-intervention |
