# Supplementary material for: Assessing the Implementation and Effectiveness of the Electronic Patient-Reported Outcome Tool for Older Adults With Complex Care Needs: Mixed Methods Study
Source: J Med Internet Res. 2021 Dec 2;23(12):e29071. doi: 10.2196/29071 (PMC8726765; doi:10.2196/29071)
Supplement: Multimedia Appendix 4 [file jmir_v23i12e29071_app4.docx]

# Multimedia Appendix 4: Sample Interview Guide Questions

**Sample questions from patient interview guide**

Can you tell me a bit about your relationship to your provider of care team at [FHT]?

Tell me about using ePRO to help you work with your provider or team to set and work on goals.

What was it like using the ePRO tool? Sample probe: What were some of the challenges you experienced?

**Sample questions from provider interview guide**

What has it been like to use ePRO tool in your approach to care?

How did you find the tool itself? Sample probe: How about in terms of how you use information to help in decision-making?

What organizational supports would be needed to help adopt this tool?

**Sample questions from manager interview guide**

Tell us a bit about the culture at your organization.

Thinking about how your clinic cares for patients with complex needs, what would a tool like this need to accomplish in order to be valuable to your organization?

What were some of the challenges you faced as an organization in implementing this tool?
