# Supplementary material for: Assessing the Implementation and Effectiveness of the Electronic Patient-Reported Outcome Tool for Older Adults With Complex Care Needs: Mixed Methods Study
Source: J Med Internet Res. 2021 Dec 2;23(12):e29071. doi: 10.2196/29071 (PMC8726765; doi:10.2196/29071)
Supplement: Multimedia Appendix 5 [file jmir_v23i12e29071_app5.docx]

# Multimedia Appendix 5: Summary of how patients and providers understood, engaged with and reflected on the adoption of the ePRO tool as aligned with Normalization Process Theory constructs.

| ***Coherence*** ^a^ | | | |
| --- | --- | --- | --- |
| **Patient summary** | **Exemplary quote** | **Provider summary** | **Exemplary quote** |
| - Coherence for patients was related to their personal beliefs around setting and achieving goals, their expectations that ePRO could make a difference to their lives, and if they had a clear goal or aim. Several disconfirming examples suggest that these individual specifications of meaning may not be sufficient, and rather communal specification may be more important, which is demonstrated through observation data. - For example, two individuals self-identified as goal oriented would suggest greater likelihood of longer-term use. Only one was a long-term user and reported a strong relationship with their provider and high confidence with regard to their goal (site A, ID08). The other, who had just met their provider and reported lower confidence with regard to their goal ended up being a short-term user (site B, ID13). | "It’s (SMART goal) clear, you know, it makes you focus more. It does make you focus more, like any type of goal setting will make you focus more, you think about it more, you know? And…you may question things more, you may do a little more research, you may change your habits like I’m trying to get to bed earlier, because I tend to push it and stay up late and still get up, you know, fairly early. " (site C, super user, ID01) | - The ePRO tool was considered coherent when it aligned to the philosophy of providers and their approach to goal-oriented care, and when it was perceived to fit “the right” patient. Coherence to the tool for providers was higher earlier in the trial when they were more engaged with the tool during the onboarding process. - Adding technology to “enable” a process requires strong alignment to coherence with that process, not just from a formative standpoint but from a normative one as well (contextual integration point). | “I did like that it flowed with the way we think in terms of our level of care and making it very patient-centered. And they’re smart goals and very achievable and things like that. So I did like that it meshed with our philosophy.” (site A, Registered dietitian, nutrition counseling) |
| ***Cognitive participation*** ^b^ | | | |
| **Patient summary** | **Exemplary quote** | **Provider summary** | **Exemplary quote** |
| - All patients engaged initially in the program, but only those who demonstrated stronger belief in ePRO (stronger coherence) continued use. Other influential factors included: being tech savvy, functionality of the tool and relationships with their provider. - Notably, when patients saw the role of the provider as pivotal to their understanding of the tool (coherence) and when that went away they discontinued use. | “(Our relationship) has got closer. Being open, being able to say what you’re feeling, what you’re thinking.” (site A, ID08, long-term user) | - Cognitive participation was highest during the goal-setting process where coherence was highest for all case sites. However, as the intervention progressed toward collective action a disconnect between how the tool related to the model of care emerged. For providers who viewed GOC as an ongoing collaborative process, the tool continued to made sense (higher coherence). However, for providers who viewed GOC as a self-management process only, provider did not see the value of the tool after the initial set up when their job was perceived to be finished (lower coherence). | “Once we’ve collaborated and figured the goals out together. The rest I’ve sort of left it up to the patients to dictate how they want to pursue it afterwards. Rather than me kind of bringing it up, or me following it or measuring certain outcomes based on [ePRO]. I primarily use it just for the goal setting aspect.” (site B, Registered dietitian)  “And so, once I felt like, OK. I checked in a few times – especially at the beginning, you know two weeks and four weeks or whatever, it may be a phone call – and then I felt like if they were comfortable with it and they knew where to find me, I kind of just let them do their thing.” (site A, [Physical Therapist], ID06) |
| ***Collective action***^c^ | | | |
| **Patient summary** | **Exemplary quote** | **Provider summary** | **Exemplary quote** |
| - Observation notes suggest that stronger relationships between patients and providers led to better onboarding experiences and stronger coherence with the understanding of the purpose of the tool. Reported as more collaborative and smoother goal setting processes in the onboarding observations. Notably contextual integration for high user patients occurred when they were able to find ways to make the tool a part of their day. There is evidence here as well that patient users were more engaged in the activity through production and reproduction of the practice of use. Likely as interactions with providers became further away, there was a loss of perceived “collective action” and that may have led to a drop of in the perceived meaningfulness and subsequent use. | "I mean if it's just for self-monitoring, which is what I began to think it was, that's one thing. But that wasn't how I had pictured it initially...I would think of it differently if I thought it was self-monitoring. I mean the business of why didn't you meet your goals. I would enter a different thing if I thought it was for my own use, I wasn't trying to explain it to some anonymous person that I never heard back from. That's what I never understood, whether there was somebody out there that was going to speak back to me or not" (site B, ID12, nonuser) | - Contextual integration was the challenge for providers with regard to the actual use of the tool (integrating into other tools), and their approach to chronic disease management (language, and self-management approach). Some providers who did see the alignment to chronic disease management wanted reminders to improve activation of the intervention – and some established new processes so they could continuously engage with the tool. - Relationships were also fundamental to support collective action. Provider site A ID06 notes “people need to know that you care and that you’ve got their back.” – the app being nonresponsive actually upends this relational piece which may not have impacted their relationship with the providers, but certainly did with the tool. | “No, it did kind of fizzle out, right, so that in the start up – well, it took a while to get it going and then once we did, I think it was good but then yeah, the challenges are with the patients, right? I’m not going to be calling and bugging them because it’s out of my work – like it wasn’t something – it’s their goals, their … up to them to do.” (site A, Registered Nurse, seniors health) |
| ***Reflexive monitoring*** ^d^ | | | |
| **Patient summary** | **Exemplary quote** | **Provider summary** | **Exemplary quote** |
| - For those intrinsically motivated toward their goal it did not seem important whether they were using the tool or not, for those less motivated by the goals, however, they judged the tool to be less valuable, particularly if they were not receiving any feedback (or additional external motivation). Notably when the tool was no longer judged to be coherent for the patient they disengaged. This also relates back to individual and communal appraisal. | “The phone, I didn’t use at all. I used the computer and that was much simpler. Because I wasn’t putting [my responses] in regularly and you weren’t getting it, I couldn’t really tell what I was doing. Like, I couldn’t see the outcome of what I was doing because I wasn’t putting it in regularly enough. when you couldn’t put it in regularly...That was my big downfall, so I never really got to use it as a tool terribly much.” (site A. ID03, nonuser) | - Providers engaged in regular reflexive monitoring throughout the implementation of the tool often relating their cognitive participation and collective action around the tool to their coherence of the tools value to their work. A number of providers perceived the tool helped with accountability and motivation for patients. - This process slowed throughout the study as providers engaged less and less with the tool and had fewer opportunities to assess its value. This is most evident when exploring the changes in provider accounts of value and impact of the tool in midterm interviews as compared with end of intervention interviews. | “Well just to see how motivated she is, the fact that she felt that she was being accountable for her own health and her activity, and then to support her and encourage her to sustain that, and just to re-assess if needed, right?” (site C, Provider ID03) |

^a^Coherence: beliefs, behaviors and acts that shape an activity as meaningful. Occurs at both the individual (individual specification) and group level (communication specification) and is produced and reproduced through ongoing interaction.

^b^Cognitive participation: individuals actively participating with the intervention.

^c^Collective action: where groups start to normalize the process and work together, involved collective purpose aimed at a shared goal. Contextual integration, linking the intervention to existing structures and procedures enables this process.

^d^Reflexive monitoring: individual and group judgments regarding the utility and effectiveness of the new practice. The formality and intensity of the monitoring work signals the level of embeddedness.
