# Supplementary material for: Assessing the Implementation and Effectiveness of the Electronic Patient-Reported Outcome Tool for Older Adults With Complex Care Needs: Mixed Methods Study
Source: J Med Internet Res. 2021 Dec 2;23(12):e29071. doi: 10.2196/29071 (PMC8726765; doi:10.2196/29071)
Supplement: Multimedia Appendix 6 [file jmir_v23i12e29071_app6.docx]

# Multimedia Appendix 6: Types of goals and tasks set during the trial

| **Stated goals** | **Permutations (at goal level)** | **Tasks** |
| --- | --- | --- |
| *Self-management goals* | | |
| 1. Physical activity | Attend activity (group/class) | YMCA physical activity programs |
|  | Walking | Walking |
|  | Exercise | Neck and shoulder |
|  |  | Treadmill |
|  |  | Walking |
|  |  | Cycling |
|  | Consistent/increase activity | Walk with friend |
|  |  | Swimming |
|  |  | Attend classes and groups |
|  |  | Increase length of walk |
|  |  | Pain management |
|  |  | Treadmill walking |
| 1. Weight loss | Weight loss | Return to COPD group |
|  |  | Daily walk |
|  |  | Food prep |
|  |  | Healthy eating |
|  |  | Exercise for blood sugar and pain management |
| 1. Nutrition | Calorie counts | Schedule denturist appointment |
|  |  | Attend weekly weight in at CCC |
|  | Mindful eating | Avoid night time snacking |
|  | Nutrition | Hydration |
|  |  | Sugar intake |
|  | Immune health via nutrition | Improve breakfast meals |
| 1. Sleep |  | Sleep |
| 1. Smoking cessation | Quit smoking | Attend clinic |
|  | Smoking cessation | Result nicotine patch |
| 1. Disease management | Improve blood sugar | Track blood sugar levels |
|  |  |  |
|  |  | Walking |
|  | Maintaining diabetes control/ Diabetes | Mindful of glucose control |
|  |  | BG monitoring |
|  |  | Reduce evening snacking |
|  | Improving skin condition | Medical diagnosis/treatment |
| 1. Physical symptom management | Pain | Manage pain |
|  | Managing migraines | Managing without medication |
| *Social goals* | | |
| 1. Attend group/programs | Attend YMCA programs | Physical activity groups |
| *Mental health goals* | | |
| 1. Mental health related | Depression | Colouring |
|  | Improve management of stress | Identify a stress management strategy |
| *Holistic/whole person goals* | | |
| 1. Well-being and independence | Time for me | Reduce screen time |
|  |  | Read a book |
|  | Get off home oxygen | Weight management |
|  |  | Exercise |
|  |  | Schedule appointment with dietitian |
| 1. Overall health (not classified at goal level) | Improve health | Eat breakfast |
|  |  | Maintain contact with smoking nurse |
|  |  | Staying off cigarettes |
|  | Staying healthy | Aerobic exercise |
|  | Managing new health issues that arise | Tracking new issues |
|  | Preventative health | Dermatologist for skin survey |
